# Pulmonary fibrotic response to aspiration of multi-walled carbon nanotubes

**DOI:** 10.1186/1743-8977-8-21

**Published:** 2011-07-22

**Authors:** Robert R Mercer, Ann F Hubbs, James F Scabilloni, Liying Wang, Lori A Battelli, Sherri Friend, Vincent Castranova, Dale W Porter

**Affiliations:** 1 Pathology and Physiology Research Branch, HELD, NIOSH, Morgantown, WV, USA; 2 Department of Physiology and Pharmacology, West Virginia University, Morgantown, WV, USA

## Abstract

**Background:**

Multi-walled carbon nanotubes (MWCNTs) are new manufactured nanomaterials with a wide spectrum of commercial applications. To address the hypothesis that MWCNTs cause persistent pulmonary pathology, C57BL/6J mice were exposed by pharyngeal aspiration to 10, 20, 40 or 80 μg of MWCNTs (mean dimensions of 3.9 μm × 49 nm) or vehicle. Lungs were preserved at 1, 7, 28 and 56 days post- exposure to determine the potential regions and target cells for impact by MWCNT lung burden. Morphometric measurement of Sirius Red staining was used to assess the connective tissue response.

**Results:**

At 56 days post-exposure, 68.7 ± 3.9, 7.5 ± 1.9 and 22.0 ± 5.1 percent (mean ± SE, N = 8) of the MWCNT lung burden were in alveolar macrophages, alveolar tissue and granulomatous lesions, respectively. The subpleural tissues contained 1.6% of the MWCNT lung burden. No MWCNTs were found in the airways at 7, 28 or 56 days after aspiration The connective tissue in the alveolar interstitium demonstrated a progressive increase in thickness over time in the 80 μg exposure group (0.12 ± 0.01, 0.12 ± 0.01, 0.16 ± 0.01 and 0.19 ± 0.01 μm for 1, 7, 28 and 56 days post-exposure (mean ± SE, N = 8)). Dose-response determined at 56 days post-exposure for the average thickness of connective tissue in alveolar septa was 0.11 ± 0.01, 0.14 ± .02, 0.14 ± 0.01, 0.16 ± 0.01 and 0.19 ± 0.01 μm (mean ± SE, N = 8) for vehicle, 10, 20, 40 and 80 μg dose groups, respectively.

**Conclusions:**

The distribution of lung burden was predominately within alveolar macrophages with approximately 8% delivery to the alveolar septa, and a smaller but potentially significant burden to the subpleural tissues. Despite the relatively low fraction of the lung burden being delivered to the alveolar tissue, the average thickness of connective tissue in the alveolar septa was increased over vehicle control by 45% in the 40 μg and 73% in the 80 μg exposure groups. The results demonstrate that MWCNTs have the potential to produce a progressive, fibrotic response in the alveolar tissues of the lungs. However, the increases in connective tissue per μg dose of MWCNTs to the interstitium are significantly less than those previously found for single-walled carbon nanotubes (SWCNTs).

## Background

Carbon nanotubes (CNTs) are a key type of nanomaterial being developed and used in manufacturing processes. Single and multi-walled carbon nanotubes (SWCNTs, MWCNTs) are two of the main tubular nanoscaled structures formed from carbon atoms. Both SWCNTs and MWCNTs are relatively durable, have a high strength-to-weight ratio, and a number of additional qualities making them desirable for a variety of manufacturing and medical applications. In evaluating the potential for pulmonary toxicity, the low reactivity of the rolled nanotube form of the graphene sheets and the corresponding low toxicity of the macroscopic graphite form would suggest a low toxicity, at least for the purified forms which do not contain the metal catalysts used in synthesis of the nanotubes. Indeed, the high mechanical stability, the ability to be functionalized and the apparent biocompatibility of CNTs has lead to the aggressive development of CNTs for tissue engineering applications [1].

However, studies with purified SWCNTs have demonstrated potentially significant health risks from pulmonary exposure [2-6]. Doses in these studies were over a wide range and included inhalation studies resulting in low lung burdens which are relevant to potential occupational exposure [4]. Subsequent studies with highly pure MWCNTs have demonstrated a inflammatory time-course and fibrotic response which are qualitatively similar to that of the SWCNT studies [6-12]. To date, direct comparisons of the effects of SWCNTs versus MWCNTs by the same route of administration and methods have been limited to a few studies [3,4,12,13]. In general, the quantitative comparisons of these studies have been focused on analysis of inflammatory mediators and/or cells in bronchoalveolar lavage. Recent cell culture studies indicate that dispersed SWCNTs may directly stimulate fibroblast proliferation and collagen production [14], suggesting that migration of CNTs to the alveolar interstitium may be a critical step in CNT-induced fibrogenesis.

While both single and multi-walled forms of CNTs have been demonstrated to have fibrotic potential, evaluations of the fate of CNT following lung exposure have frequently been limited to reports of visual impressions. These indicate that SWCNTs and MWCNTs differ significantly in how the material is distributed within the lungs. Quantitative distribution determinations have been made for SWCNTs [3-5]. These results demonstrate that SWCNT distribution in the alveolar region is comprised of two components the ratio of which depends on the initial dispersion state. In studies where pure, but relatively poorly dispersed material are used, large clumps of SWCNTs from 5 to 20 microns are initially found in the lungs [2,5]. These clumps accounted for approximately 80% of the lung burden in our prior studies and were found to rapidly induce the development of large granulomatous lesions within the first 7 days of exposure [5]. The remaining 20% of the lung burden was found to be in single nanotubes, nanoropes or small, submicron structures that were found to be rapidly incorporated into the alveolar interstitium. Macrophage phagocytosis of the pure, SWCNT form was not a significant component. A subsequent study using a highly dispersed form of pure SWCNTs reported that SWCNTs induced interstitial fibrosis but did not contain large clumps of SWCNT and did not produce granulomatous lesions [3]. These observations would indicate that the fibrotic response of the lungs is due to the dose of SWCNTs delivered to the alveolar interstitium in the submicron form.

MWCNT lung burden is much more diversely distributed between cells and/or alveolar regions. In general the MWCNT form of CNTs is more easily dispersed before administration, and the number and size of granulomatous lesions that form from the agglomerates are significantly fewer and smaller than those present in SWCNT-exposed lungs [15]. In lungs exposed to MWCNTs, the CNTs are found to be distributed in every cell/cell layer of the lung parenchyma [12]. Indeed, a substantial number of nanotubes are found, within hours of exposure, distributed in the subpleural interstitium and penetrating through the visceral pleura into the pleural space [13]. Unlike SWCNTs, alveolar macrophages in MWCNT-exposed lungs are observed to be highly loaded with nanotubes [6,7,12,13]. Although the distribution of MWCNTs differs significantly from that of SWCNTs, MWCNT-exposed lungs still demonstrated interstitial fibrosis [12]. However, the qualitative nature of the histopathologic evaluation of MWCNT-exposed lungs to date precludes a direct quantitative comparison of the fibrotic response between the two forms of nanotubes.

In this study, we sought to determine the fraction of the dose of MWCNTs delivered to the alveolar interstitium and, thus, determine if the inherent potential of MWCNTs to produce an interstitial fibrotic response differs significantly from that of SWCNTs when exposure was conducted via similar methods in the same laboratory. To accomplish this, we determined the distribution of MWCNT lung burden to alveolar macrophages, the alveolar interstitium, and other compartments at different doses and times and quantified the degree of alveolar fibrotic response.

## Results

Tests for potential redistribution of MWCNT fibers during sectioning did not demonstrate any tendency for MWCNT fibers to be redistributed. MWCNT fibers in the subpleural tissue region averaged 4.7 ± 0.6 and 5.1 ± 0.7 (mean ± SE, N = 16) fibers per half lobe profile in the top and bottom counts, respectively. There was no statistically significant difference in the number of MWCNT fibers in the subpleural tissue in the top versus the bottom. This is consistent with a prior study which specifically focused on MWCNTs in the pleura [13], with high magnification imaging of MWCNTs in the same sections as the present report. The FESEM and light microscopy of that study, involved detailed examination of hundreds of MWCNTs in the subpleural/pleura region at high resolution but did not produce any evidence of redistributed fibers.

**Figure 1:**
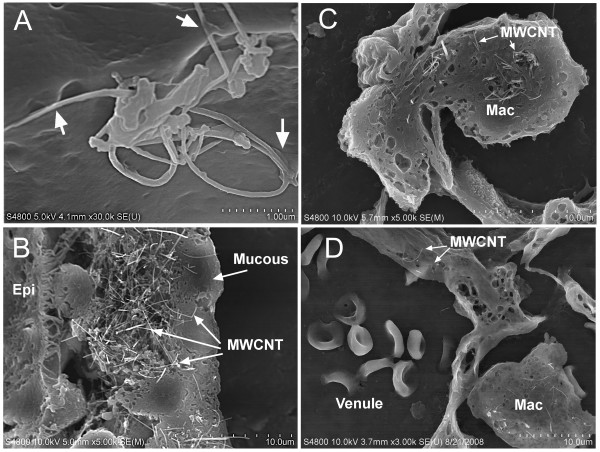
**FESEM examination of lungs**. A high magnification image of MWCNTs on the alveolar epithelial surface is shown in Figure 1A. Arrows indicate numerous points where the MWCNTs are being enveloped by the Type I alveolar epithelium 1 hour after aspiration. Figure 1B shows MWCNTs in the mucous blanket of an airway 1 hour after aspiration. A MWCNT-loaded alveolar macrophage (Mac) is shown passing through the alveolar wall in Figure 1C (28 days post-aspiration, 80 ug dose). MWCNTs penetrating through the endothelial wall into the lumen of a pulmonary venule are indicated by the arrows of Figure 1D (28 days post-aspiration, 80 ug dose).

Field emission scanning electron microscope (FESEM) examination of the lung surface demonstrated that MWCNTs were being rapidly incorporated in the alveolar epithelium (Figure 1A) and mucous lining layer (1B). MWCNTs were observed in the mucous lining layer at early time points of 1 hour and 1 day post-aspiration. MWCNTs in the airways at these early time points were rapidly cleared as demonstrated by their absence in the airways at 7, 28 or 56 days after aspiration. At all time points of the study, MWCNT-loaded alveolar macrophages, such as the one illustrated in Figure 1C, were the most prominently observed foci of CNTs. In examination of the fibers caught in section, such as Figure 1C, intracellular MWCNTs did not appear to be associated with intracellular vacuoles and many MWCNTs were found penetrating the cell membrane and/or completely passing through the cell. This suggests that either 1) normal phagocytosis may not be the central mechanism for loading of alveolar macrophages with MWCNTs or 2) phagocytized MWCNTs were not maintained within the phagolysosome. MWCNTs could be found penetrating into the vascular space of the lungs, such as the ones shown in the pulmonary venule of Figure 1D. Although these cases of vascular penetration were rare, they suggest some degree of transport of MWCNTs out of the lungs via the circulation.

**Figure 2:**
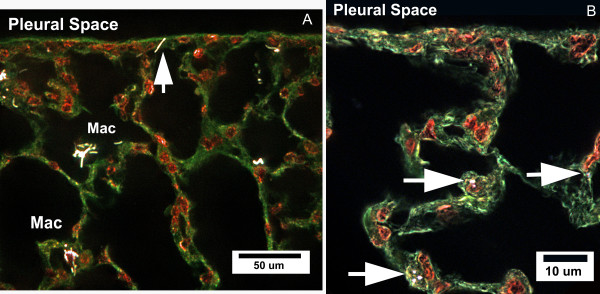
**Enhanced darkfield image of CNT-exposed lungs**. Figure 2A shows the general distribution of MWCNTs in the lungs 7 days after aspiration (40 ug dose) with the section oriented with the pleural space running along the top. CNTs scatter light with high efficiency and thus produce the bright, white structures in these enhanced darkfield images, while nuclei (red) and other tissues (green) produce a significantly duller image. Arrow points to an individual MWCNT penetrating into the mesothelial cell layer forming the boundary between the alveolar tissues and pleural space. While alveolar macrophages are foci for MWCNTs, scattered, submicron MWCNT structures can be found in the alveolar interstitium throughout the section. Figure 2B gives a comparison image from a mouse lung exposed to a highly dispersed preparation of SWCNTs (aspiration 10 ug dose, 7 day). In the case of dispersed SWCNTs, the majority of CNT structures are rapidly incorporated into the alveolar interstitium (arrows).

The enhanced dark field images of Figure 2 illustrate the general alveolar distribution of MWCNTs (Figure 2A) and highlight the similarities and differences from that of dispersed SWCNTs from a previously reported study [3] (Figure 2B). Examination of sections from MWCNT-exposed lungs demonstrates that this form of CNTs readily penetrates all cell membranes/boundaries of the lungs. While MWCNTs are found be highly concentrated in alveolar macrophages, they have also been found to rapidly penetrate the alveolar epithelium and visceral pleura [13]. In contrast, SWCNTs are seldom found in alveolar macrophages, but appear to predominately migrate through the alveolar epithelium to the interstitial space [3]. As illustrated in Figure 2, the MWCNT and SWCNT structures, which distribute to the interstitial space, are submicron in dimension and are comparable in size between the two forms of CNTs.

**Figure 3:**
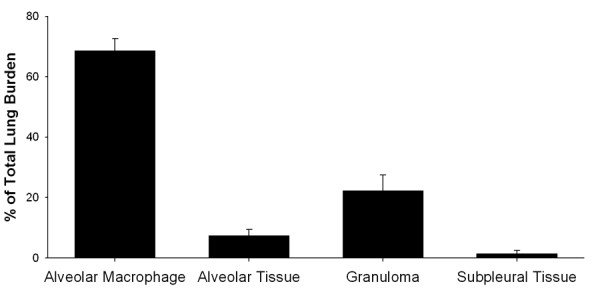
**Morphometric determination of the lung distribution of MWCNTs 56 days post- aspiration**. Results show the distribution of MWCNT burden in airways, alveolar and subpleural tissue regions of the lungs 56 days after aspiration of an 80 ug dose. Results are expressed as a percentage of the total lung burden. As shown in the graph, MWCNTs within, partially within or completely penetrating alveolar macrophages account for the majority of the MWCNT lung burden. Data are means ± SE for an N of 8 animals per group.

The lung burden distribution of MWCNTs in alveolar macrophages, the alveolar interstitium, granulomas in the airspaces and subpleural tissue regions at 56 days post-aspiration (80 μg dose) are shown in Figure 3. The results demonstrate the prominent incorporation of MWCNTs into alveolar macrophages. The majority of the MWCNTs were found within and/or penetrating into alveolar macrophages (68% of the total lung burden). MWCNTs in the interstitium of the alveolar tissue accounted for 8% of the total lung burden at this time. Subpleural tissue, the region consisting of mesothelial cells of the visceral pleura and immediately adjacent interstitium, contained 1.6% of the total lung burden. MWCNTs were rarely observed in the airways at 7, 28 or 56 days post-aspiration.

**Figure 4:**
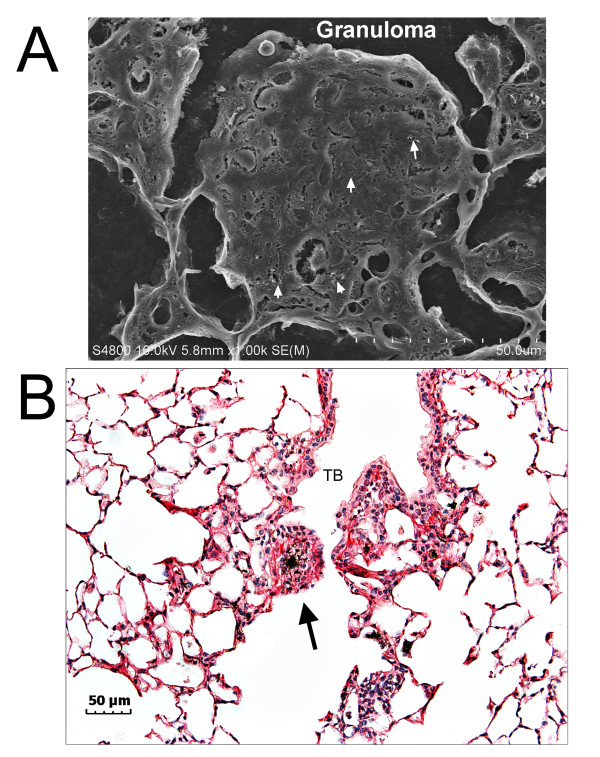
**FESEM and light micrographs of fibrotic granulomas following MWCNT aspiration**. The FESEM image of Figure 4A shows a granulomatous lesion which is essentially filling an alveolus of the lung (40 ug dose, 56 days). Arrows indicate MWCNTs in the section of the granuloma. The light micrograph in Figure 4B illustrates the extensive collagen network (red) developed to encircle a small mass of MWCNTs in the airspace (black, 20 μg dose, 56 days). Collagen fibers are red in the section due to staining with Sirius Red. TB-terminal bronchiole.

Granulomatous lesions in the alveolar airspace accounted for 20% of the lung burden. A representative FESEM image of the granulomatous lesions formed by MWCNTs at 56 days after aspiration of a 40 μg dose is shown in Figure 4A, and a light microscopic image of another lesion (arrow) is shown in Figure 4B. These granulomatous lesions were relatively rare compared to those found in SWCNT- exposed lungs [2,5] but when present were comparable to the size of an alveolus. Generally, the granulomas were located in or near the alveolar region immediately proximal to the terminal bronchiole as shown in Figure 4B.

**Figure 5:**
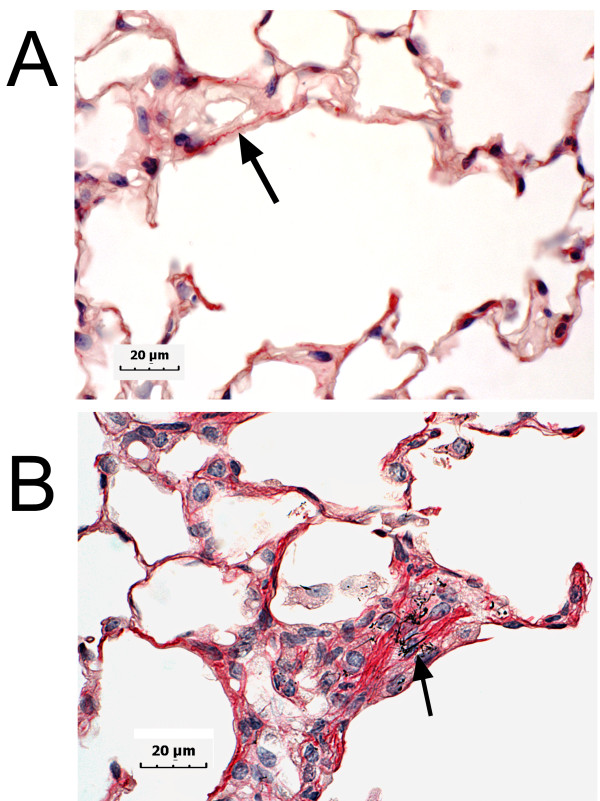
**Light micrograph of fibrotic response to interstitial MWCNTs**. Comparision of Sirius red staind collagen fibers in PBS treated (5A) and MWCNT treated lungs (5B, 80 ug dose at 56 days post-aspiration). Figure 5A illustrates the typical long and thin collagen fibers which traverse within the alveolar interstitium of a normal lung. MWCNTs incorporated into the alveolar interstitium were found to be associated with significant interstitial development of collagen fibers and accompanying interstitial cells. The region of the alveolar wall containing the MWCNTs (arrows) is substantially thicker than the average alveolar wall thickness of 4 to 5 um which is typical of a normal mouse. Sirius Red-Hematoxylin stained sections.

A representative light micrograph of the interstitial fibrotic response to MWCNTs 56 days after aspiration of an 80 ug dose is shown in Figure 5B with a comparison of a PBS aspiration treated lung section in Figure 5A. Single fibers and isolated small clumps of well-dispersed MWCNTs can be found throughout the abnormally thickened alveolar wall in the center of the micrograph (Figure 5B). MWCNTs were not present in the much thinner and more normal appearing alveolar walls in the upper left of the micrograph. As described in the Methods section, mean linear intercept for the alveolar airspace was determined. This measure of the average free distance of the alveolar airspaces was 26.1 ± 0.3, 27.8 ± 0.9, 26.9 ± 0.3, 27.5 ± 0.3 and 27.3 ± 0.5 um (mean ± SE, N = 8) for the PBS control and 10, 20, 40 and 80 ug dose groups, respectively. There were no statistically significant differences in the mean linear intercept between groups indicating that the focal thickening of the alveolar wall illustrated in Figure 5 was not sufficiently global to alter alveolar airspace dimensions.

**Figure 6:**
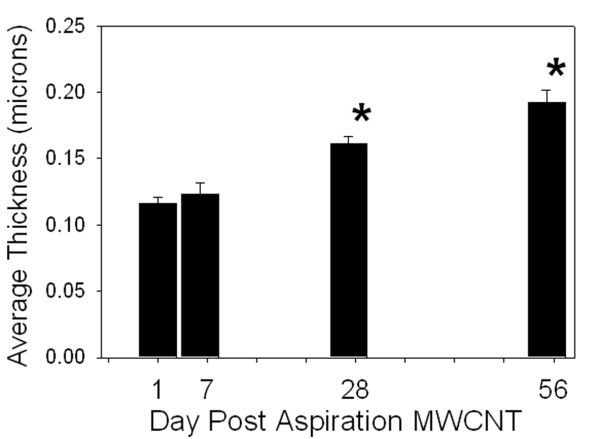
**Morphometric determination of the time-course of collagen response day 1 to day 56 post-exposure to an 80 ug MWCNT dose**. Results from morphometric determination of Sirius Red stained alveolar interstitial collagen fibers expressed as the average thickness of connective tissue in the alveolar interstitium at various times after aspiration of MWCNTs. At 28 days, there was significant accumulation of connective tissue fibers in the alveolar wall which continued to increase through 56 days after aspiration. Day 1 and day 7 post-exposure were not significantly different from PBS-treated controls. Data are means ± SE for an N of 8 animals per group. * indicates statistical significance P < 0.05 vs. DM -treated control mice.

The corresponding graph of the morphometrically determined changes in alveolar collagen fibers, identified by Sirius Red staining, expressed as the average thickness of connective tissue fibers at various times following aspiration of the 80 ug dose is given in Figure 6. This graph demonstrates a progressive increase in thickness of connective tissue in the alveolar interstitium over time, which was statistically significant by day 28 post-exposure. On average, connective tissue thickness in the alveolar region increased by 1.2% per day at the 80 ug dose. As indicated in the methods, collagen accumulation in the lungs due to granulomatous lesions was determined separately. Connective tissue fibers in the granulomatous lesions of the airspaces expressed as a percentage of the total connective tissue fibers in the alveolar regions of the lungs was 0.5 ± 0.2, 2.8 ± 0.8 and 7.0 ± 1.2% (mean ± SE, N = 8) at days 7, 28 and 56 post-exposure, respectively.

**Figure 7:**
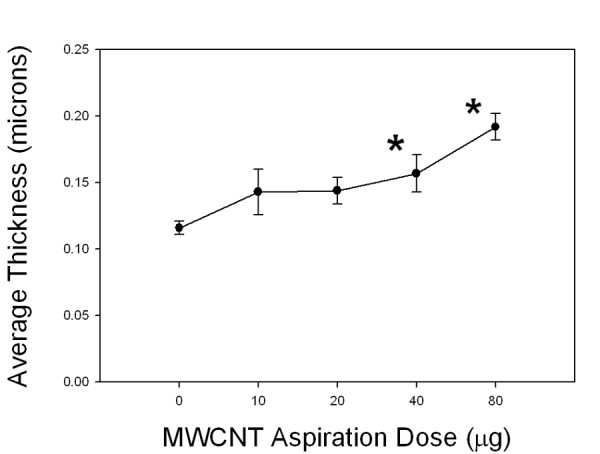
**Collagen dose-response at 56 days after exposure to MWCNTs**. As shown in Figure 7, there was a progressive increase in the average thickness of the alveolar connective tissue thickness versus aspiration dose of MWCNTs. At a dose of 80 ug the average thickness was increase by 73% over control. Data are means ± SE for an N of 8 animals per group. * indicates statistical significance P < 0.05 vs. DM -treated control mice.

Results from the analysis of the dose-response between aspirated dose of MWCNTs and the thickness of alveolar connective tissue 56 days after exposure are given in Figure 7. The progressive increase in the average thickness of alveolar connective tissue versus aspiration dose of MWCNTs was approximately linear with an r^2 ^of 0.95. The slope was equal to an increase of alveolar connective tissue thickness by approximately 1% for each microgram of MWCNT dose above control. At a dose of 80 ug the average thickness of connective tissue fibers stained by Sirius Red was increased by 73% over control.

## Discussion

Pulmonary responses to MWCNT exposure include an acute inflammatory phase, a progressive fibrotic response in the interstitium of the alveolar wall, and a granulomatous inflammation enveloping any airspace deposits of MWCNT agglomerates [6,10,12,16]. The acute inflammatory reaction peaks at 7 days in the mouse and resolves, albeit at a slow pace as judged by lavage analysis, over a 56 day period [12]. MWCNTs accumulating at the bronchoalveolar junction can cause a granulomatous response to airspace deposits of MWCNTs resulting in infiltration and encasement by epithelioid macrophages to form a connective tissue rich nodule that "walls-off" the MWCNTs in the airspace and is a typical foreign body response of the lungs to insoluble and/or poorly cleared particles [17-19]. In addition to these components, which have demonstrated toxic responses for MWCNTs, the pleural region and the lymphatics may be a site of toxic responses to MWCNTs as reports have shown the presence of MWCNTs in the subpleural tissue after inhalation and aspiration exposures [8,12,13,20].

Understanding of the mechanisms responsible for and importance of these varied responses to MWCNTs is complicated by the widespread distribution of MWCNTs throughout the lung parenchyma. The widespread distribution of MWCNTs in virtually every major cell and region of the lung parenchyma makes determination of the distribution of MWCNTs in the lungs necessary in order to evaluate sensitivities of the different regions to MWCNT exposure. In the present study, we sought to determine the distribution of MWCNT burden in the lungs and examine how the dose to the alveolar interstitium altered the fibrogenic response. Such analysis allowed the sensitivity to the interstitial dose for MWCNTs to be compared to SWCNTs, thus determining relative fibrogenic potency.

Although composed of the same carbon-carbon bond arrangement, the diameter and rigidity of MWCNTs and SWCNTs differ significantly. Individual SWCNTs are 1 to 4 nm in diameter and typically several hundred nanometers in length [5]. Individual MWCNTs used in this study are more fiber-like with mean length of 3.9 um and 49 nm diameter [12]. At this fundamental level, SWCNTs are comparable to the thread-like filaments observed in basement membranes of the lungs [21,22]. MWCNTs are more similar in dimensions to the collagen fibrils forming the collagen fibers of alveolar interstitium [23]. For SWCNTs, the actual structures observed in exposed lungs do not correspond to a single CNT fundamental unit. As we have shown previously with TEM and confocal examinations [3], SWCNTs are rapidly incorporated into the interstitial space with few being incorporated in alveolar macrophages. SWCNTs are rarely found as individual nanotubes. The more typical interstitial form of SWCNT incorporation is as submicron groupings of nanotubes similar to those shown in Figure 2B. The mode of lung exposure does not seem to be a factor as no difference in the distribution of SWCNTs was found comparing aspiration versus short term (4 day) inhalation exposure to well dispersed SWCNTs [4].

As shown in the present study, MWCNT incorporation into the alveolar interstitial spaces is also a rapid process with individual nanotubes or small clumps or nanotangles of several poorly organized nanotubes being most commonly observed. This pattern was observed in lungs preserved within 1 hour of aspiration (Figure 1) as well as many days after exposure (Figures 1, 2 and 5). This indicates that there is no significant redistribution once the nanotubes are incorporated into the alveolar interstitium. Of note, the fraction of the MWCNT lung burden incorporated into the alveolar interstitium of approximately 8% is significantly lower than that of well-dispersed SWCNTs where upwards of 90% of the lung burden is deposited in the alveolar septa (Table 1). This difference in alveolar interstitial lung burden between MWCNTs and SWCNTs is mainly due to the large portion of lung burden carried by alveolar macrophages in the case of MWCNTs.

**Table 1:** Sensitivity of Interstitial Fibrotic Response to SWCNTs versus MWCNTs

	SWCNTs^1^	MWCNTs
**Aspiration Dose** **(ug)**	**10 ug**	**80 ug**

**Lung Burden Distribution** **(%)**	**10% Alveolar Macrophage** **90% Interstitial**	**70% Alveolar Macrophage** **8% Interstitial** **20% Airspace Granuloma** **2% Subpleural Tissue**

**Alveolar Interstitial Dose** **(ug)**	**9 ug**	**6.4 ug**

**Connective Tissue Thickness (um)**	**0.10 to 1.10** **Control Exposed**	**0.11 to 0.19** **Control Exposed**

**Sensitivity** **Δum Thickness/Δug Dose**	**0.11 um/ug**	**0.013 um/ug**

^1 ^Data for dispersed SWCNT interstitial response from Mercer et al. [3]

A comparison of the fibrotic response between SWCNTs and MWCNTs would appear to suggest that SWCNTs are significantly more fibrogenic than MWCNTs. For instance, Table 1 gives the connective tissue thickness increase from 0.1 um in PBS controls to 1.1 um in mice with an aspiration dose of 10 ug of well-dispersed SWCNTs at 28 days post-exposure [3]. In contrast, in the present study of MWCNTs, Table 1 shows that the average thickness of connective tissue increased from 0.1 um in PBS controls to 0.2 um at the 80 ug aspiration dose. Clearly, when compared on the basis of mass delivered to the lungs, the alveolar interstitial fibrotic response to MWCNTs is significantly lower than that of SWCNTs. In part these differences in response are accounted for by differences in the alveolar interstitial dose for these two types of CNTs (Table 1). For SWCNTs, 90% of the lung burden is distributed to the interstitium whereas only 8% of the MWCNTs lung burden is distributed to the interstitium, thus the lower alveolar interstitial dose of the MWCNTs. The lower MWCNT dose to the alveolar interstitium results in a lower response. A lower fibrogenic activity of MWCNTs than SWCNTs has been reported with *in vitro *studies measuring proliferation and collagen production of lung fibroblasts [14,24,25].

When the target site doses are used to assess the sensitivity of the alveolar interstitium to CNT exposure, it is found that SWCNTs induce a change in connective tissue thickness of 0.11 um/ug of interstitial dose while the corresponding value for MWCNTs is 0.013 um/ug. After accounting for the dose delivered to the critical target site of the alveolar interstitium, SWCNTs are found to be approximately 8.5-fold more fibrogenic than MWCNTs. A recent NIOSH Current Intelligence Bulletin [26] suggests that MWCNTs and SWCNTs may differ by 7.5-fold in adverse health effects based on analysis of exposure levels to carbon nanotubes associated with a 10% increase in abnormal response (BMD-benchmark dose). In the NIOSH analysis, BMDs of 3.6 and 0.48 ug/lung were reported for MWCNTs and SWCNTs exposures of rodent lungs, respectively, using data from the Porter et al. [12] and the Shvedova et al. [4] studies.

Comparison of response between MWCNTs and SWCNTs, on the basis of mass as the denominator may not be the most relevant basis as the fibrotic reaction is likely due to either a toxic species formed by a chemical reaction on the surface of the CNT or interaction of the CNTs with cell surfaces and/or interior organelles. The dose, expressed in terms of surface area of CNT structures, is thus an alternative means of comparing the CNT response that should be considered. Surface area measurement by nitrogen gas adsorption/desorption is the most commonly reported characterization of nanoparticle surface. As indicated in the Methods section, the BET based surface of MWCNTs was 26 m^2^/g. Shvedova et al.[4] reported a surface area of 508 m^2^/g for SWCNTs. The BET measure of surface area would indicate a 20-fold greater surface area per microgram of SWCNTs than MWCNTs in the lungs assuming that the two CNTs were dispersed to the same degree. Using this result as a basis to evaluate the relative response of connective tissue change to dose, expressed as surface area, would indicate that MWCNTs are approximately 2.5 times more potent than SWCNTs. Uncertainty in what the actual responsive surfaces areas are, i.e. the surface area of the delivered structure, for either MWCNTs or SWCNTs in the interstitium of the lungs prevents accepting this factor of 2.5 fold as being accurate. However, it does indicate that differences in responsive surface areas between MWCNTs and SWCNTs may account for the differences in connective tissue response. A direct stimulation of fibrogenesis by SWCNTs and MWCNTs is supported by in vitro studies which indicate that CNT fibers directly induce proliferation and collagen production by lung fibroblast [14,25].

In spite of the quantitative differences in alveolar fibrotic response to SWCNT and MWCNT the general pulmonary response is qualitatively similar, resulting in transient inflammation but persistent granulomatous lesions and fibrosis. In mice exposed to CNT by pharyngeal aspiration (10 μg/mouse), SWCNT caused a greater inflammatory response than MWCNT at 1 day post exposure [3,5,12]. Morphometric analyses indicate that well- dispersed SWCNT are not well recognized by alveolar macrophages (only 10% of the alveolar burden being within alveolar macrophages), while 90% of dispersed SWCNT structures rapidly cross alveolar epithelial cells and enter the interstitium [3]. In contrast, as shown in this study, 68.7% of MWCNT in the respiratory zone enter alveolar macrophages and 7.5% migrate into the alveolar interstitium. This difference in pulmonary fate of SWCNT versus MWCNT is likely to partially account for the quantitative differences in alveolar fibrotic response. The reason for this difference in macrophage uptake is unknown. Lastly, SWCNT exhibit a greater fiber count per mass than MWCNT. Therefore, on an equal mass lung burden basis more SWCNT interact with the lung than with MWCNT. Since fibrotic potency in the present study usead a mass dose metric, this may explain part of the potency difference. At present, there is no satisfactory method to determine the number of fibers per mass for SWCNT versus MWCNT.

## Conclusions

In this study, we determined the distribution of MWCNTs following lung exposure as well as the dose and time relationships to connective tissue response of the alveolar interstitium. The majority of the MWCNT lung burden was initially and chronically in alveolar macrophages. The alveolar interstitium received approximately 8% of the MWCNT lung burden. In addition, FESEM and enhanced dark field microscopy clearly demonstrate that the CNT clumps delivered to the interstitial space were comparable in size for both SWCNTs and MWCNTs. Airways contained MWCNTs at 1 hour and 1 day after exposure but were cleared of MWCNTs at 7 days and later times.

MWCNTs were found to produce a time and dose dependent increase in collagen content of the alveolar interstitium. After accounting for the fraction of lung burden delivered to the interstitium, interstitial collagen response to MWCNT exposure was found to be significantly less than that of SWCNT on a mass delivered basis. When compared on the basis of CNT surface area delivered to the interstitium the relative responses are more nearly equal. The results demonstrate that there are differences in the fibrotic response between the two forms of carbon nanotubes that cannot be explained by the mass of CNT distributed to the interstitial space. This suggests that surface active reactions may be involved in the interstitial response(s) to SWCNT and MWCNT. This mechanism is supported by in vitro studies indicating that CNT can directly stimulate proliferation and collagen productions by lung fibroblasts [14,24,25].

## Methods

### Animal

Male C57BL/6J mice (7 weeks old) were obtained from Jackson Laboratories (Bar Harbor, ME). Mice were housed one per cage in polycarbonate ventilated cages, which were provided HEPA-filtered air, with fluorescent lighting from 0700 to 1900 hours. Autoclaved Alpha-Dri virgin cellulose chips and hardwood Beta-chips were used as bedding. Mice were monitored to be free of endogenous viral pathogens, parasites, mycoplasms, Helicobacter and CAR Bacillus. Mice were maintained on Harlan Teklad Rodent Diet 7913 (Indianapolis, IN), and tap water was provided ad libitum. Animals were allowed to acclimate for at least 5 days before use. All animals used in this study were housed in an AAALAC-accredited; specific pathogen-free, environmentally controlled facility. All animal procedures were approved by the NIOSH ACUC.

### Carbon Nanotube Source

MWCNTs used in this study were obtained from Mitsui & Company (XNRI MWNT-7, lot #05072001K28) and were fully characterized in a previous report [12]. Briefly, MWCNT trace metal contamination was 0.78%, with sodium (0.41%) and iron (0.32%) being the major metal contaminants. Average MWCNT surface area measured by nitrogen absorption-desorption technique (Brunauer-Emmett-Teller method, BET) was 26 m^2^/g. MWCNT median length was 3.86 μm and count mean diameter was 49 ± 13.4 (mean ± S.D.) nm, as determined by scanning electron microscopy of MWCNTs suspended in dispersion medium as described below [12].

### Pharyngeal Aspiration of MWCNTs

Suspensions of MWCNTs for aspiration were prepared in a dispersion medium (DM) as reported previously [15]. Dispersion medium was made up of Ca^+2 ^and Mg^+2 ^phosphate-buffered saline (PBS), pH 7.4, supplemented with 5.5 mM D-glucose, 0.6 mg/ml mouse serum albumin and 0.01 mg/ml 1,2 dipalmitoyl-sn-glycero-3-phosphocholine (DPPC). DPPC was prepared fresh as a 10 mg/ml stock solution in absolute ethanol. DM has been shown to be an effective dispersing agent for MWCNTs and does not elicit toxicity or mask surface reactivity [15,27].

Mice were exposed to MWCNTs by pharyngeal aspiration as described previously [12]. Briefly, mice were anesthetized with isoflurane (Abbott Laboratories, North Chicago, IL) for pharyngeal aspiration of DM (vehicle control) or 10, 20, 40 or 80 μg of MWCNTs. In a previously reported study [12] the 10 ug dose in mice was shown to approximate human deposition for a person performing light work for between 9 months and 7.5 years based on average daily MWCNT workplace exposure data.

When fully anesthetized, the mouse was positioned with its back against a slant board and suspended by the incisor teeth using a rubber band. The mouth was opened, and the tongue gently pulled aside from the oral cavity. A 50 μl aliquot of sample was pipetted on the base of the tongue, and the tongue was restrained until the droplet was aspirated by at least 2 deep breaths (but for not longer than 15 seconds). Following release of the tongue, the mouse was gently lifted off the board, placed on its left side, and monitored for recovery from anesthesia. This alternative to inhalation has been shown to produce a even distribution of fluorescent particles throughout the lung [28]. Recently published direct comparisons studies between inhalation and aspiration of SWCNTs have further demonstrated the applicability of the aspiration technique for such studies [4].

### Body Weights

Body weights were 21.1 ± .2, 22.5 ± .8, 23.5 ± .5 and 26.5 ± .8 grams for 1, 7, 28 and 56 day groups given an aspiration dose of 80 ug MWCNT (mean ± SE, N = 8). Body weights for day 1 and day 56 DM aspiration groups were 21.9 ± .7 and 26.9 ± .7 grams (mean ± SE, N = 8). There were no significant differences in initial body weight or weight gain between MWCNT exposed and DM aspiration groups.

### Lung Fixation and Section Preparation

At 1, 7, 28 and 56 days after aspiration, mice were euthanized by an overdose of pentobarbital (> 100 mg/kg body weight, i.p.) followed by transection of the abdominal aorta to provide exsanguination. Seven to 8 animals were studied at each time point. The lungs were fixed by intratracheal perfusion with 1 ml of 10% neutral buffered formalin. To accomplish lung fixation, the trachea was cannulated and the lungs removed from the chest cavity. The lungs were then inflated with 1 ml of 10% neutral buffered formalin over a 1 minute period and the trachea tied off. After 4 to 5 hours the lungs were trimmed and processed overnight in a tissue processor. For each animal, the left lung lobe were placed in the embedding carrier with a consistent apex to base orientation and embedded in paraffin.

For morphometric studies, paraffin sections of the left lung (5 μm thick) were cut. A new region of the disposable knife blade was used to section each block and the water bath was changed frequently in order to prevent potential cross-contamination that might result from MWCNT passage on the knife between sections. The sections were then deparaffinized and rehydrated with xylene-alcohol series to distilled water. To enhance the contrast between tissue and MWCNTs, lung sections were stained with Sirius Red [29]. Sirius Red staining consisted of immersion of the slides in 0.1% Picrosirius solution (100 mg of Sirius Red F3BA in 100 ml of saturated aqueous picric acid, pH 2) for 1 - 2 hours followed by washing for 1 minute in 0.01 N HCl. Sections were then briefly counterstained in freshly filtered Mayer's hematoxylin for 2 minutes, dehydrated, and mounted on a slide with a coverslip. Additional sections were stained with hematoxylin and eosin for routine pathology assessment as previously reported [12].

Due to the fiber-like nature of MWCNT, the potential for fibers to be redistributed over the section by the cutting blade was a concern. To test for this possibility a count was made of MWCNT fibers in the subpleural tissue region in the top half of a section versus MWCNT fibers in the subpleural tissue region in the bottom half of the sections. The distinction of top versus bottom being made based on the direction of the sectioning blade through the block. This comparison was made on 16 lung profiles from 1 day post MWCNT exposed mice at the 80 ug dose. Half of the blocks had the apical portion of the lungs at the bottom of the block when sectioned and the other half were reversed to eliminate possible bias due to apical/basal gradients of lung burden. Analysis of this tissue provided no evidence of fiber redistribution dut to blade sectioning.

### Field Emission Scanning Electron Microscopy

For scanning electron microscopy, sections of the lung were cut at 8 microns, placed on carbon planchets, deparaffinized and sputter coated. After coating, the specimens were examined with a Hitachi Model S-4800 Field Emission Scanning Electron Microscope (FESEM) at 5 to 10 kV and working distances of 4.5 mm to 6 mm for magnifications of 100,000× to 1000×, respectively. Photographs were taken in slow scanning mode at 1280 × 1024 pixels. Use of thin sections from paraffin embedded tissue was found to be preferable to large, unevenly cut blocks because it provided a uniform thickness of organic material on the carbon planchet. The 8 micron sections were thick enough to convey three-dimensional information but were also less likely to charge or undergo physical shifts when examined at the high magnifications necessary to study nanomaterials.

### Enhanced-Darkfield Light Microscopy Imaging of Nanoparticles

Carbon nanotubes in sections from exposed lungs were assessed using an enhanced-darkfield optical system. Nanomaterials, such as carbon nanotubes, have dimensions less than the wavelength of light, have closely packed atoms, and typically have a refractive index significantly different from that of biologic tissues and/or mounting medium. These characteristics produce significantly greater scattering of light by nanoparticles than by the surrounding tissues. The enhanced-darkfield optical system images light scattered in the section and, thus, nanomaterials in the section stand-out from the surrounding tissues with high contrast. Using this method of imaging, lung sections can be easily scanned at relatively low magnification to identify CNTs that would not be detected by other means.

The optical system consisted of high signal-to-noise, darkfield-based illumination optics adapted to an Olympus BX-41 microscope (CytoViva, Auburn, AL 36830). Sections for dark-field examination were specifically cut from paraffin blocks and collected on ultrasonically cleaned, laser cut slides (Schott North America Inc, Elmsford, N.Y. 10523) to avoid nanoparticle contamination from the ground edges of traditional slides. After staining with Sirius Red-Hematoxylin, sections were coverslipped with Permount. After alignment of the substage oil immersion optics with a 10× objective, sections were examined with 60× air or 100× oil immersion objectives. Enhanced darkfield images were taken with a 2048 × 2048 pixel digital camera (Dage-MTI Excel digital camera XLMCT, Michigan City, In 46360).

### Lung Distribution of MWCNTs

The distribution of MWCNTs in the lungs was determined by counting the occurrence of MWCNTs under an eyepiece point counting overlay using standard morphometric point counting methods [30] as previously described for study of the distribution of SWCNTs [3]. Point counting categories were subdivided into points over MWCNTs in airway region, points over MWCNTs in alveolar regions, and points over MWCNTs in the subpleural tissue region. Airway regions were defined as those containing airway tissue (airway epithelial cells-basement membrane and tissues of the broncho-vascular cuff), airway lumen, and associated blood vessels greater than 25 microns. Alveolar regions were those containing alveolar tissue and alveolar air space. The subpleura tissue region included MWCNTs in the subpleural tissue and MWCNTs in the visceral pleural surface. The subpleural tissue regions included the immediately subpleural alveolar interstitial-epithelium layer and subpleural lymphatics but did not include any portion of alveolar walls attaching to the pleura. Points in airway and alveolar regions were further subdivided into points over MWCNTs that were in the airspace, points over MWCNTs that were in tissue of the region, and points over MWCNTs that were partially or completely within macrophages.

To accomplish the counting, an eyepiece counting overlay consisting of 11 by 11 lines (121 total points for each throw of the overlay) was used with a 100× oil immersion objective. A grid pattern for throws of the counting overlay was used in order to insure a uniform sampling of the section which did not overweight interior points. The counting overlay throws of the eyepiece were positioned over the section at 12 uniformly spaced grid points in both × and Y co-ordinates. These 12 grid points were determined using the stage micrometer scale to measure the × and Y bounds of the section. Using the bounding rectangle of these co-ordinates, a 3 by 4 grid was selected and the 12 intersections were used as the center point for each of the eyepiece counting overlay throws.

For each animal, three sections were counted and the counts for the airways, alveolar and subpleural tissue regions were summed. Each counting category was divided by this total and multiplied by 100 to express the results as a percentage of total lung burden. Eight animals were analyzed per group.

### Morphometric Analysis of Collagen Distribution

Collagen fibers in the lungs were detected with Sirius Red staining [29], which has been demonstrated to be a quantitative morphometric method for collagen fiber determination in the lungs [31,32]. Quantitative morphometric methods were used to measure the average thickness of Sirius Red positive connective tissue fibers in the alveolar regions. Volume and surface density were measured using standard morphometric analyses [33,34]. This consisted of basic point and intercept counting. Volume density was determined from counting the number of points over all tissues in the alveolar regions and points over Sirius Red positive connective tissue. Surface density of the alveolar wall was determined from intercepts between a line overlay and the alveolar wall. These point and intercept counts were made using a 121-point/11-line overlay graticule (12.5 mm square with 100 divisions), at 100× magnification, taken at six locations equally spaced across each section (one section per animal). This process was repeated twice for each animal. In order to limit the measurements to alveolar parenchyma, areas containing airways or blood vessels greater than 25 mm in diameter were excluded from the analysis. Average thickness of the Sirius Red positive connective tissues fibers of the alveolar wall was computed from two times the ratio of volume density of point to the surface density of the alveolar wall. The collagen fiber content of granulomatous lesions in the airspaces was assessed by a separate tabulation of points over Sirius Red positive connective tissues in granulomas and expressed as a percentage of total alveolar collagen. Mean linear intercept, a measure of the average size of the alveolar/alveolar duct airspaces in the alveolar region, was computed from the ratio of volume density to surface density [33].

### Statistical Analyses

Data were analyzed using analysis of variance (STATGRAF). Bartlett's test was used to test for homogeneity of variances between groups. Statistical differences were determined using one-way analysis of variance with significance set at p ≤ 0.05. When significant F values were obtained, individual means were compared to DM-treated controls using Duncan's multiple range test [35] and P < 0.05 was considered to be significant. Data are given as means ± SE.

## Abbreviations

BET: Brunauer-Emmett-Teller method: BMD: benchmark dose: CNT: carbon nanotubes; DM: dispersion medium; DPPC: 1,2 dipalmitoyl-sn-glycero-3-phosphocholine; FESEM: field emission scanning electron microscope; MWCNTs: multi-walled carbon nanotubes; PBS: phosphate-buffered saline; SWCNTs: single-walled carbon nanotubes; TEM: transmission electron microscope.

## Competing interests

The authors declare that they have no competing interests.

## Authors' contributions

RM conceived of the study, developed the morphometric methods, conducted the FESEM evaluation, analyzed the experimental results and drafted the manuscript. AH was involved in the planning and writing of the manuscript. JS performed the morphometric counting and assisted in analysis of results. LW contributed to the experimental design and assisted in lung preparation. LB provided important information on sampling of the lungs for study and conducted lung preparation for histopathology. SF assisted in the sampling design and operation of the CytoViva studies. VC and DP contributed to the experimental design, acquisition of funding and writing of the manuscript. All authors read and approved the final manuscript.
